# High-Tech Methods of Cytokine Imbalance Correction in Intervertebral Disc Degeneration

**DOI:** 10.3390/ijms241713333

**Published:** 2023-08-28

**Authors:** Natalia A. Shnayder, Azamat V. Ashhotov, Vera V. Trefilova, Maxim A. Novitsky, German V. Medvedev, Marina M. Petrova, Ekaterina A. Narodova, Daria S. Kaskaeva, Galina A. Chumakova, Natalia P. Garganeeva, Natalia V. Lareva, Mustafa Al-Zamil, Azat R. Asadullin, Regina F. Nasyrova

**Affiliations:** 1 Institute of Personalized Psychiatry and Neurology, Shared Core Facilities, V.M. Bekhterev National Medical Research Centre for Psychiatry and Neurology, 192019 Saint Petersburg, Russia; ashkhotov.v@mail.ru (A.V.A.); vera.v.trefilova@yandex.ru (V.V.T.); 2 Shared Core Facilities “Molecular and Cell Technologies”, V.F. Voino-Yasenetsky Krasnoyarsk State Medical University, 660022 Krasnoyarsk, Russia; stk99@yandex.ru (M.M.P.); katya_n2001@mail.ru (E.A.N.); dashakas.ru@mail.ru (D.S.K.); 3 Department of Neurology, Hospital for War Veterans, 193079 Saint Petersburg, Russia; maximnovitsky93@gmail.com; 4 R.R. Vreden National Medical Research Center for Traumatology and Orthopedics, 195427 Saint-Petersburg, Russia; dr.medvedev.g@yandex.ru; 5 Department of Therapy and General Medical Practice with a Course of Postgraduate Professional Education, Altai State Medical University, 656038 Barnaul, Russia; g.a.chumakova@mail.ru; 6 Department of General Medical Practice and Outpatient Therapy, Siberian State Medical University, 634050 Tomsk, Russia; garganeeva@gmail.com; 7 Department of Therapy of Faculty of Postgraduate Education, Chita State Medical Academy, 672000 Chita, Russia; larevanv@mail.ru; 8 Department of Physiotherapy, Faculty of Continuing Medical Education, Peoples’ Friendship University of Russia, 117198 Moscow, Russia; alzamil@mail.ru; 9 Department of Psychiatry and Addiction, Bashkir State Medical University, 450008 Ufa, Russia; droar@yandex.ru; 10 International Centre for Education and Research in Neuropsychiatry, Samara State Medical University, 443016 Samara, Russia

**Keywords:** intervertebral disk degeneration, inflammation, treatment, high-tech method, genetic technology, cell technology

## Abstract

An important mechanism for the development of intervertebral disc degeneration (IDD) is an imbalance between anti-inflammatory and pro-inflammatory cytokines. Therapeutic and non-therapeutic approaches for cytokine imbalance correction in IDD either do not give the expected result, or give a short period of time. This explains the relevance of high-tech medical care, which is part of specialized care and includes the use of new resource-intensive methods of treatment with proven effectiveness. The aim of the review is to update knowledge about new high-tech methods based on cytokine imbalance correction in IDD. It demonstrates promise of new approaches to IDD management in patients resistant to previously used therapies, including: cell therapy (stem cell implantation, implantation of autologous cultured cells, and tissue engineering); genetic technologies (gene modifications, microRNA, and molecular inducers of IDD); technologies for influencing the inflammatory cascade in intervertebral discs mediated by abnormal activation of inflammasomes; senolytics; exosomal therapy; and other factors (hypoxia-induced factors; lysyl oxidase; corticostatin; etc.).

## 1. Introduction

**Figure 1:**
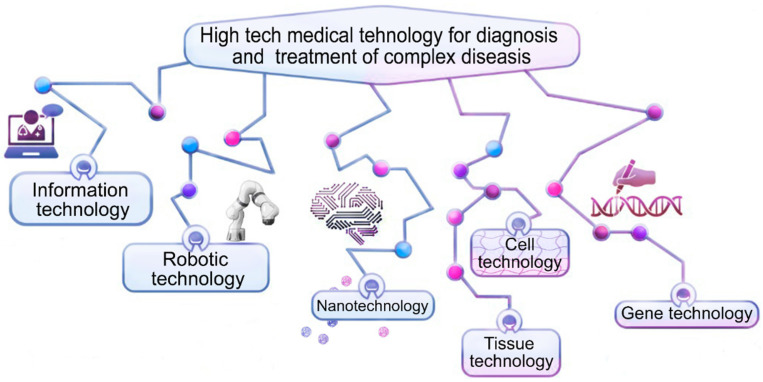
High-tech methods of treatment of complex diseases.

**Figure 2:**
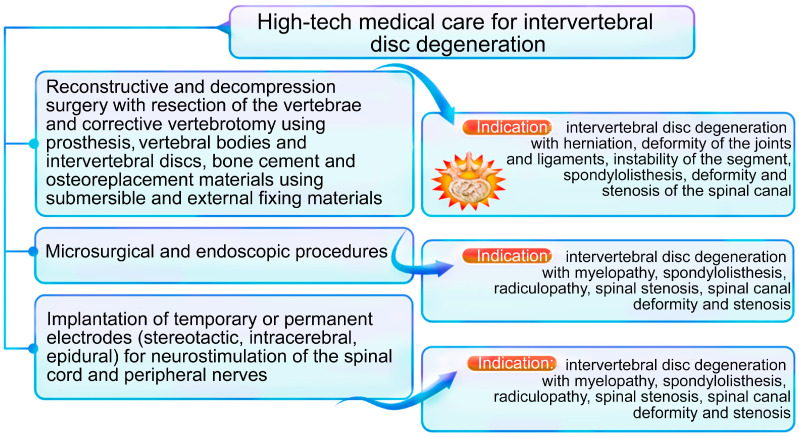
High-tech methods of treatment of intervertebral disc degeneration and indications for their use.

High-tech medical care (HTMC) is a part of specialized care and includes the use of new and unique methods of treatment (resource-intensive, and as a rule, with proven effectiveness) [1]. HTMC can be provided for a number of profiles, including: vertebrology, traumatology, orthopedics, neurosurgery, etc. [1]. HTMC is used in a wide range of human diseases, which are characterized by complex mechanisms of pathogenesis and a progressive type of course, as well as when classical and new approaches to therapy are not effective enough or are not effective at all (Figure 1). Severe intervertebral disc degeneration (IDD) and the progressive type of IDD are indications for the use of high-tech therapies [2] (Figure 2).

**Figure 3:**
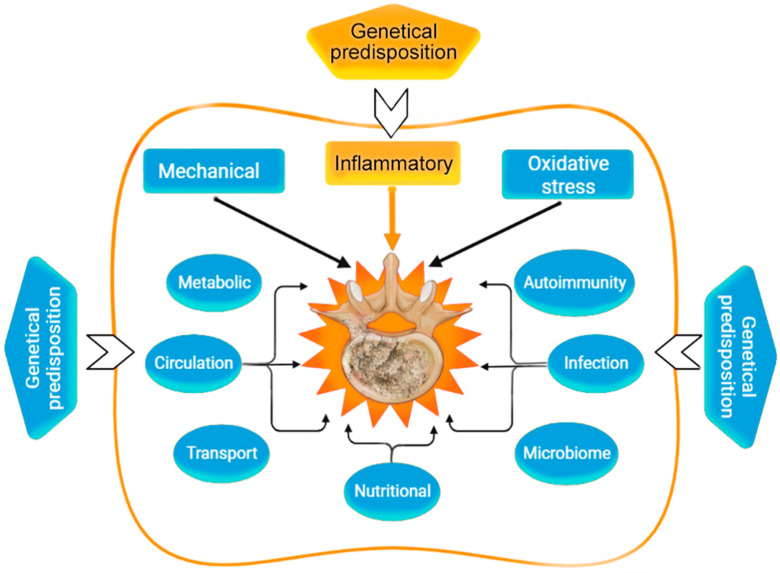
Risk factors of intervertebral disc degeneration in humans [3].

It is likely that IDD begins to develop in adolescence and progresses at a variable rate, leading to the formation of degenerative or traumatic herniated intervertebral discs (IVDs), chronic back pain, and disabling complications (spinal canal stenosis, spinal root compression, paresis, paralysis, etc.). The mechanisms of development and progression of IDD continue to be studied, but so far, there is no single point of view [3] (Figure 3). In recent years, cytokine imbalance became considered as one of the important pathogenic mechanisms for IDD, which affects the risk of developing persistent vertebrogenic back pain, progressive apoptosis of chondrocytes, and other cells of the nucleus pulposus (NP) and fibrous ring (AF). Knowledge about the molecular mechanisms of acute and chronic inflammation and the role of these in degeneration of IVDs, based on a critical analysis of preclinical and clinical studies of the last decade, was presented by us in a previous publication [3].

Inflammatory response is viewed as a negative mechanism for severe degenerative damage to IVDs. It is likely that a balanced inflammatory response is needed to reduce the risk of development and rate of IDD progression, as well as for the most complete (most possible for a particular patient) restoration of the function of damaged IVDs. Cytokine imbalance affects the overall effect of chronic inflammation in patients with IDD and the expected therapeutic response to pharmacotherapy [4]. In turn, pharmacotherapy of a cytokine imbalance in IDD conducted by prescribing both well-known (“classical”) and new drugs [4] can have various effects, including: (1) an anti-inflammatory effect (directing a protective immune response in patients with IDD in the direction of reducing chronic inflammation); and (2) a regenerative effect (directing a protective immune response in patients with IDD towards healing). 

**Figure 4:**
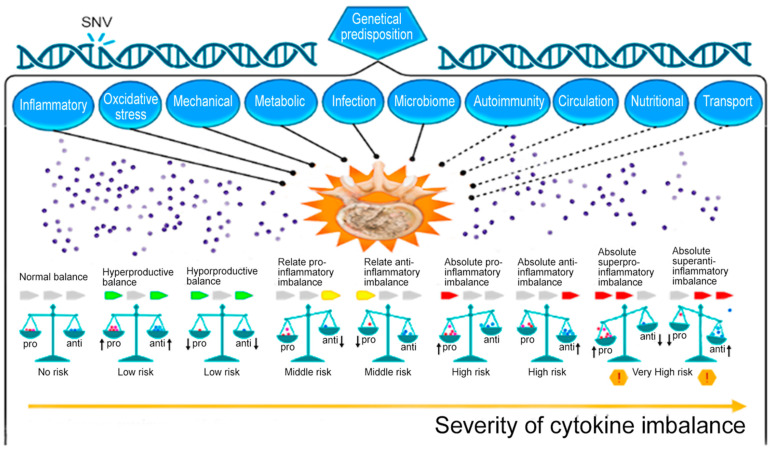
Possible variants of cytokine balance disorders in patients with intervertebral disk degeneration (IDD) [3]. Note: SNV—single-nucleotide variant; anti—anti-inflammation cytokines; pro—pro-inflammation cytokines; solid line—well-studied mechanisms of IDD; dotted line—insufficiently studied mechanisms of IDD; down arrow—decreased cytokine level; up arrow—increased cytokine level; and red exclamation point in a yellow hexagon—a dangerous situation.

Unfortunately, well-known classical approaches and new approaches to the management of IDD do not always give the expected therapeutic response. The attention of scientists and clinicians is drawn to genetic, cellular, and other methods of HTMC for cytokine imbalance correction in patients with severe IDD. At the same time, the absolute high-producing cytokine imbalance (as possible variant of cytokine balance disorders in patients with IDD) may be most unfavorable in terms of IDD progression rate and the formation of severe chronic back pain (Figure 4), as we discussed earlier [3]. It is probably the leading indication for the use of HTMC methods (Figure 2) in adult patients with IDD, who lack the expected therapeutic response to classical and new methods of pharmacotherapy and surgery [4]. Such new methods, in particular, and persistent inflammatory processes in general in patients with IDD, are of undoubted scientific and clinical interest. However, we did not find any previously published narrative reviews on this topic.

The aim of the review is to update knowledge about new high-tech methods based on cytokine imbalance correction in IDD.

## 2. Promising High-Tech Methods for Correcting Cytokine Imbalance in Intervertebral Disc Degeneration

**Figure 5:**
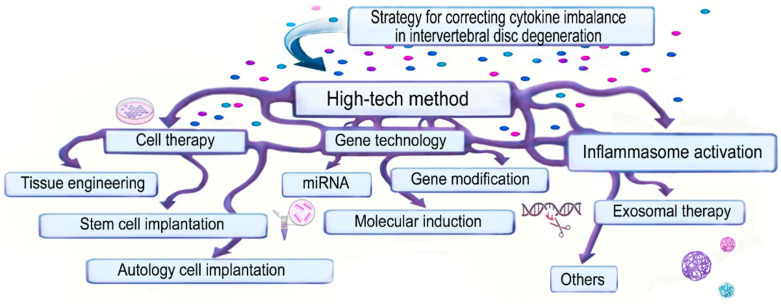
High-tech methods for correcting cytokine imbalance: the figure demonstrates that these methods can be used as a separate method, and as a combination of methods (arrow interlacing).

The use of HTC techniques for cytokine imbalance correction in IDD is a new promising direction in the non-surgical treatment of this disease in adults, but not in children. However, indications for their use in adolescents may be extended, since, as is known, the onset of degenerative processes in NP and AF begins much earlier than the onset of clinical symptoms of IDD. Figure 5 presents promising HTMC methods for correcting cytokine imbalance in IDD, which are proposed for use both in the form of independent (separate) and combined methods; for example, stem cell implantation (independent method) or a combination of stem cell implantation with exosome therapy (combined method).

## 3. Cell Therapy

### 3.1. Stem Cell Implantation

Mesenchymal stem cells (MSCs) are considered as a source of cells for gene therapy and implantation. It is known that MSCs are non-committed pluripotent stem cells that are present in various tissues. They are available and easy to manipulate [5], but MSCs must be differentiated into chondrocyte-like cells before implantation in patients with IDD. Growth factors of the family of bone morphogenetic proteins are used to differentiate MSCs into chondrocytes [6]. In recent years, a differentiation factor from the cyclooxygenase (COX) family was studied as a more specific factor for the differentiation of MSCs, and members of the Brachyury transcription factor family are considered as MSCs adhesion factors [7]. The cultivation of MSCs with IVD cells is used to induce a disk-like phenotype [8], and cultivation of MSCs under conditions of a three-dimensional system promotes the formation of a chondrocyte-like phenotype [9].

Increasingly, MSCs are being used in clinical trials for severe IDD and severe low back pain in humans. The mechanisms of action of MSCs are not fully understood. However, they are known to reduce the levels of pro-inflammatory cytokines in the pro-inflammatory/degenerative microenvironment of IVDs [10]. Preconditioning of MSCs with IL-1β increases the secretion of hIL-6, hIL-8, hMCP-1 (monocyte chemoattractant protein 1), and other pro-inflammatory biomarkers of IDD. On the other hand, MSCsec suppress the expression of genes encoding bIL-6, bIL-8, and metalloproteinase 1 (MMP-1). In contrast, MMP-3 and the tissue inhibitor of metalloproteinase 2 (TIMP-2) increase the expression of these genes. Increased aggrecan deposition was found in MSCsec-treated degenerating IVDs, although no differences were observed in other EMC components. Protein analysis of MSCsec-treated IVD supernatant revealed a significant increase in chemokine ligand 1 (CXCL-1), MCP-1, macrophage inflammatory protein 3 alfa (MIP-3α), IL-6, IL-8, and growth-related oncogenes alfa/beta/gamma (GRO-α/β/γ) and decreased interferon gamma (IFN-γ), IL-4, IL- 5, IL-10, and TNF-α. At the same time, MSCsec-treated IVD supernatants did not stimulate neo-angiogenesis and neurogenesis in vitro [11]. The immunomodulatory paracrine effect of MSCs in IDD (without a clear effect on extracellular matrix remodeling) was shown in the course of studies and suggests that the mechanism of action of MSCs depends on the cytokine feedback loop [12]. Delivery of MSCs to degenerating IVDs increases the population of Tie2-positive progenitors (differentiation clusters) and prevents apoptosis of NP and AF cells. Additionally, they may induce a proliferative response in NP and AF cells of degenerative IVDs [13]. Human umbilical cord-derived MSCs and chondroprogenitor derivatives may reduce back pain, inflammation, and promote cell regeneration in IVDs in a rat model of IDD [14]. Lithium chloride preconditioning (medium level) mechanically induced an increase in cellular reactive oxidative species (ROS) and ERK-1/2 (extracellular signal-regulated kinase 1/2) activation, which is closely associated with increased cell and ECM survival in IVDs. Treatment with preconditioned adipose-derived stem cells (ADSCs) showed a better therapeutic effect than control transplantation of ADSCs, with better preservation of NP cells and deposits of ECM in degenerative IVDs [15].

### 3.2. Implantation of Autologous Cultured Cells

Degenerated IVDs can be populated with in vitro cultured cells. To prevent immune reactions, such cells must be autologous. The use of NP material selected at the time of microdiscectomy may be one of the approaches to obtaining cells for in vitro cultivation. However, the possibility of introducing autologous cultured cells or implants after surgery in IVDs is debatable [16]. The simplest approach to repair degenerating IVD using autologous cultured cells is the injection of cells grown ex vivo [17]. This approach allows achieving a significant therapeutic response in patients with IDD.

Another approach to using autologous cells for transplantation into a degenerating IVD is to culture the cells in a 3D culture system. A sufficient number of artificial three-dimensional matrices for culturing IVD cells was proposed [18]. At the same time, Gruber et al. [19] used autologous cells cultured in a conventional monolayer culture, which were then populated in a three-dimensional matrix in accordance with the cavity formed in the degenerating IVD. At 33 weeks, NF and AF cell structures were similar to that of healthy IVDs.

### 3.3. Tissue Engineering

Cell therapy has some limitations in IDD therapy during in vitro studies, in vivo studies, and in many clinical studies [20]. Transplantation of MSCs may be an effective treatment for mild to moderate IDD. However, multifunctional tissue engineering treatment has advantages in severe IDD requiring structural support [21,22,23]. Tissue engineering (co-administration of growth factors, MSCs, and scaffold) is more important because of the positive results of using various types of functional polymers (alginate, chitosan, collagen, gelatin, hyaluronic acid, polyurethane, polyethylene glycol, and polyglycolic and polylactic acids). This high-tech method based on cells and scaffolds is considered as a more effective method for the treatment of severe IDD in humans [23]. 

Biomaterials for tissue engineering can be developed in the form of injections to mimic and preserve the structures of the IVD extracellular matrix, taking into account its degenerative changes at an early stage of the disease. Therapeutic small molecules or drugs can be incorporated into biomaterials to stop the pathological cascade that underlies the development of IDD. All of these biomaterials can be delivered to degenerating IVDs using percutaneous, minimally invasive procedures, in addition to existing treatments for early and mild disease [24]. Additionally, biomaterials can serve as an advanced cell delivery system in degenerating IVDs to support transplantation of MSCs, to repopulate native cells in IVDs with mild to severe degeneration associated with lower cellularity in IVD tissues.

A tissue engineering strategy based on precision biomaterials, taking into account the severity of the degenerative process in IVDs, is aimed at providing a regenerative effect in early, mild, and severe IDD. This is necessary to develop customized biomaterials and tissue engineering strategies to halt disease progression, stimulate NP and AF cell regeneration, and alleviate discogenic pain syndrome. Due to the advantages of an individual approach, many researchers are developing various tissue engineering methods to replace degenerating IVD in animal models [25,26]. 

## 4. Gene Technologies

### 4.1. Gene Modifications

Current gene technology for IDD therapy is based on the adeno-associated virus (AAV) gene delivery systems. However, there is a limitation (for example, immune responses to viral proteins) [27,28,29,30,31]. The knockdown-mediated self-complement AAV serotype 6 (ADAMTS-4) induces a long-term and effective increase in aggrecan synthesis in degenerating human NP cells. Self-complementary AAV (scAAV) vectors, which do not express any viral genes and are not associated with any known diseases in humans, are attractive vectors for delivering therapeutic genes to degenerative IVDs [32]. Thus, scAAV-6, scAAV-2, and scAAV-3 demonstrated high and prolonged expression of transgenic green fluorescent protein with transduction efficiency of 98.6%, 91.5%, and 89.6%. Unlike scAAV-6, scAAV-2, and scAAV-3, serotypes reduced NP cell viability by 25% and 10%, respectively. Moreover, scAAV-6 did not affect the expression of inflammation markers, ECM proteins, and the catabolic process in degenerating IVDs [33]. 

Lentivirus and AAVs are traditional viral vectors used in gene therapy for severe IDD. New genetic technologies of RNAi (RNA interference) and short palindromic repeat method (CRISPR) further enhanced their benefits. Non-viral vectors, such as polyplex micelles and exosomes, are promising vectors for IDD [34]. Editing the epigenome of inflammatory receptors with lentiviral CRISPR can be considered as a promising strategy for gene therapy for severe IDD [35].

Other viral vector-mediated gene transfers into NP and AF cells are also being actively studied, including: retrovirus; baculovirus; and lentivirus [36]. Additionally, virus-free gene transfer into IVD cells is possible: RNA interference (RNAs); targeted destruction of microbubbles by ultrasound; Micelle/Cas9 polyplex associated with cluster regulatory intervals, and short palindromic repeats (CRISPR/Cas9)) [36]. Bi et al. [36] attenuated H₂O₂-induced acute inflammation in IVD cells by overexpressing the *Klotho* gene using RNAs in a rat model of IDD and by inhibiting Toll-like receptor 4 (TLR-4). Knockdown of the *Klotho* gene with RNAs reduced anti-inflammatory and protective effects in the animal model of IDD. Accordingly, the *Klotho* gene expression possibly regulates TLR4-NF-κB signaling [37,38].

CRISPR/Cas9 is a convenient and versatile tool for genome modification. It is an accurate and effective method of gene technology for treatment of severe IDD. Additionally, CRISPR/Cas9 is easier to use than other genome editing technologies [36]. This system is used in both viral and non-viral delivery methods [39]. Regarding IDD gene therapy strategy, regulation of TNF-1 receptor (TNFR-1) and IL-1 receptor (IL1R-1) signaling by the CRISPR lentivirus epigenome editing system was tested in NP and AF cells of degenerating human IVDs to suppress overexpression of TNFR-1 and IL1R-1, and related inflammatory responses [40]. These studies demonstrated that expression of TNFR-1 was suppressed by increasing aggrecan levels and decreasing MMP-3 levels when editing the genome of the transforming granulocyte growth factor receptor type 1 (TGFR-1). However, IL1R-1 expression was not downregulated and did not show any changes in aggrecan and MMP-3 levels in degenerating IVDs after IL1R-1 genome editing [35]. The ability of the CRISPR/Cas9 genome editing system can be demonstrated through the regulation of TNFR-1 only, but not regulation of IL1R-1 [35].

The transient receptor potential vanilloid type 4 (*TRPV4*) gene was successfully knocked out in vitro using the CRISPR/Cas9 gene editing system in persons with chronic low back pain [41]. Cambria et al. investigated the role of the *TNFR4* gene in regulation of IL-6 and IL-8 expression via CRISPR and reduction in the risk of severe chronic inflammatory development caused by AF cell hyper-physiological stretching [41].

All the presented HTMC methods of gene modification can be considered as promising high-tech methods for correcting cytokine imbalance in severe IDD in humans [10]. Despite a sufficient number of studies demonstrating the flexibility of the method of direct gene delivery using virus-based vectors in NP and AF cells of degenerating IVDs, the question of which gene should be delivered remains open. Researchers’ attention is focused on genes encoding anabolic factors (tumor growth factor beta 1 (FGT-β1); latent membrane protein 1 (LMP-1); COX-9; and anti-catabolic factor TIMP-1). Potential target genes are those encoding transcription factors for MMPs, TIMPs, disintegrin, and chondrocyte-specific transcription factors [42,43], as well as key genes associated with the inflammatory response such as the *IL1B* gene [3,44].

The first studies on exogenous gene delivery in vivo were carried out by Nishida et al. [45]. The authors used an AAV carrying the *FGTB1* gene in rabbits and noted a significant increase in the expression of FGT-β31, as well as proteoglycan in IVDs. When LMP-1 was introduced into the rabbit IVD tissue in vivo, an increase in the expression of anti-inflammatory cytokines, bone morphogenetic proteins 2 and 7 (BMP-2, BMP-7), and aggrecan was observed, which confirms the expediency of using this factor as a new high-tech method for IDD in humans [46]. 

COX-9 has no effect on proteoglycan synthesis. However, when the *COX9* gene is transferred into degenerating human IVD cells, the synthesis of type II collagen increases. Transfection of the *COX9* gene in the adenoviral vector into degenerating rabbit IVD ensured preservation of the structure characteristic of unaffected IVD, while typical degenerative changes in IVD were observed in the control group of animals [47]. Additionally, the *TIMP1* gene was delivered to a degenerating human IVD using an adenovirus vector. This manipulation contributed to an increase in the content of proteoglycan in IVD cell culture [48].

Despite the positive results of the use of injection methods of IDD therapy in humans, when a toxic microenvironment is formed, there is a decrease in the supply of glucose and oxygen nutrients, as well as an “acidification” of the environment, and the question remains of how long the expression of a genetically engineered construct is possible under these conditions [16].

### 4.2. MicroRNA

There is growing evidence that many cellular processes (cell proliferation, apoptosis, and pro-inflammatory cytokine release) are regulated by a new class of small non-coding RNAs known as microRNAs (miRNAs), which are 19–25 nucleotides long [49]. MiRNAs may play an important role in various disorders (for examples, cancer, neurodegeneration, and aging) [50,51,52]. MiRNAs mediate their biological functions through base pairing with the 3′-untranslated regions (3′UTRs) of their miRNAs targets to suppress protein translation and/or induce miRNAs degradation [49]. MiRNAs make up only 1–3% of the human genome, but can regulate up to about 30% of human protein-coding genes [53]. 

MiRNAs may play an important role in IDD development. Additionally, they may be promising therapeutic targets in patients with severe IDD [54,55]. Thus, miR-34a dysregulation plays a key role in apoptosis of cartilage end plate chondrocytes and degradation of the extracellular matrix in IVD [56]. MiR-34a expression is induced by IL-1β, and miR-34a silencing can prevent IL-1β-induced suppression of type II collagen synthesis in chondrocytes. In addition, miR-34a is a GDF-5 repressor [4]. So, GDF-5 deficiency significantly reduced the expression of aggrecan and collagen type II miRNAs and the content of prostaglandins. The *GDF5* gene mutation not only altered the growth and differentiation of NP cells, reduced the synthesis of the extracellular matrix, and induced IVD degeneration, but also affected the height or length of IVDs, long bones, causing a change in their height [57]. Insertion of the *GDF5* gene has significant clinical implications for the repair of ECM in degenerated IVDs [58].

MiR-15b levels are elevated in people with IDD. Suppression of miR-15b reduced IL-1β-induced degradation of ECM structures in human NP by increasing the expression of SMAD-3 (TGFβ signal transducers) [59].

MiR-202-3p was shown to suppress IL-1β-induced MMP-1 production in degenerative IVDs. Conversely, anti-miR-202-3p treatment markedly increased MMP-1 production. In addition, mutation of the miR-202-3p binding site in the 3′-UTR of MMP-1 mRNA abolished miR-202-3p-mediated repression of reporter activity. These results suggest that miR-202-3p may regulate expression of MMP-1 in human NP and contribute to IDD development [60].

IL-6 may aggravate IDD by causing ferroptosis of cartilage cells, which is caused by inhibition of miR-10a-5p and subsequent de-repression of the IL-6R signaling pathway [61]. The IL-6/miR-10a-5p/IL-6R axis may be a new target for IDD treatment.

### 4.3. Molecular Inducers of Intervertebral Disc Degeneration

LncRNA and the taurine-activated gene 1 (*TUG1*) were involved in IDD development. Suppression of *TUG1* gene expression can protect NP cells from TNF-α-induced apoptosis and senescence while simultaneously stimulating proliferation by blocking the Wnt/β-catenin pathway [62]. LncRNA expression of the zinc finger antisense 1 (*ZFAS1*) gene is associated with increased risk and severity of IDD [63]. LncRNA HOX transcript antisense RNA (HOTAIR) promotes NP cell aging and apoptosis, as well as degradation of ECM in IVD via the Wnt/β-catenin activation pathway [64]. 

Overexpression of the X-inactive specific transcript (*XIST)* gene, which is a key effector gene for X-chromosome inactivation in mammals, contributed to IL-1β-induced NP cell degeneration. It is suggested that the *XIST* gene may also contribute to the development and progression of IDD through NP apoptosis and degradation of EMC structures [65]. MiR-499a-5p expression was lowered in NP cells in patients with IDD and could bind to XIST, while its upregulation reversed the effects of overexpression of XIST in NP cells treated with IL-1β [65]. 

MiR-34a-5p can bind to HOTAIR lncRNA and promote NP apoptosis by targeting NOTCH-1 [66]. MiR-153-3p binds to LINC00641 to suppress NP cell autophagy and promotes NP cell death [67]. MiR-326 is downregulated in IDD and is involved in the mechanisms by which LncRNA small nucleolar RNA host gene 1 (*SNHG1*) overexpression promotes NP cell proliferation [68]. MiR-499a-5p can suppress NP cell apoptosis and ECM structures degradation in IVDs by targeting COX-4 [69]. Additionally, overexpression of the *XIST* gene in NP cells can promote cellular apoptosis and degradation of EMC through targeting miR-499a-5p [65].

The *PIEZO1* gene (a mechanically sensitive ion channel gene) may play an important role in the regulation of inflammation activity of NLRP-3 (main component of the inflammasome type of the same name (NLRP-3 inflammasome)) [70]. PIEZO1-siRNA is used to suppress ex-downregulation of the *PIEZO1* gene, which inhibits the activation of the Ca/NF-κB pathway in NLRP-3 inflammasome and ultimately slows down the inflammatory response in IVDs. In addition, LINC00969 regulates the thioredoxin-interacting protein (*TXNIP*) gene transcription, acting as a competitive miR-335-3, and thereby regulates NLRP-3 inflammasome activity [4].

## 5. Technologies for Intervertebral Disc Inflammatory Cascade Mediated by Abnormal Inflammasome Activation

Inflammasome is a macromolecular protein complex that finely regulates caspase 1 activation. Additionally, it regulates IL-1β and IL-18 expression [71]. A sensitive component of the inflammasome is part of the oligomerization nucleotide-binding domain containing protein (NOD)-like receptor (NLR) proteins [72,73]

NLRP-3 senses “danger” in the form of lysosomal destabilization or a decrease in the intracellular level of K+ [72]. It is known that the conformational change in NLRP-3 occurs as a result of activation of a secondary protective mechanism (leakage of cathepsin B into the cytoplasm and outflow of K+ from the cell) in the presence of microbial or non-microbial pathogens that cannot be digested by lysosomes [72]. Such conformational changes in NLRP-3 lead to oligomerization and recruitment of the macromolecular assembly of the caspase associated with apoptosis, recruited by the domain-containing (ASC) spotted protein [72,73,74]. An important component for the formation of NLRP-3 is the enzymatic effector—caspase 1. This enzyme can perform various functions that are not related to the inflammasome, but its main function in the formation of the inflammasome is the cleavage of pro-IL-1β to the active form of the pro-inflammatory cytokine IL-1β [72,75]. 

Activation of caspase 1 inside the inflammasome leads to pyroptosis [75]. Pyroptosis is associated with cell swelling, increased permeability of the cell membrane, and cell rupture. As a result, pro-inflammatory mediators (for example, pro-IL-1α) are released into the extracellular space [72,75,76]. Damage to the cell membrane is mediated by the substrate of caspase 1—gasdermin D. It provides the formation of oligomers of N-terminal fragments inside the cell membrane after its cleavage, followed by the formation of pores [77]. Gasdermin D pores are permeable to macromolecules and mediate unconventional extracellular release of mature IL-1β, IL-18, and active caspase 1 [78].

The Nod-like receptor 1 (NLRP-1), Nod-like receptor 3 (NLRP-3), NLRC-4, Pyrin, and AIM-2 (absent in melanoma 2 protein) are known to be mainly involved in inflammasome formation [79]. As with other NLRs, NLRP-3 consists mainly of three domains: the amino-terminal pyrine domain (PYD), the NACHT nucleotide-binding domain, and the carboxy-terminal leucine-rich repeat sequence (LRR) domain [77,79]. Upon stimulation of extracellular inflammation, NLRP-3 is induced by transcription and controls post-translational modification. Activated NLRP-3 additionally recruits ASCs through PYD-PYD homotypic interactions and induces ASC assembly into large mottled structures [80].

The NLRP-3 inflammasome plays an important role in the development of IDD. So, NLRP-3 inflammasome activation and the IDD process mediated by NLRP-3 inflammasome involve the regulation of multiple signaling pathways. For example, the NF-κB pathway is mainly involved in the initiation of NLRP-3 inflammasome, but Fas-associated protein with death domain and caspase-8 are also involved in the induction of NLRP-3 inflammasome expression [81,82].

Intracellular oxidative stress, endoplasmic reticulum stress, mitochondrial dysfunction, and lysosome rupture induced by various intrinsic and extrinsic stimuli in cells of degenerating IVDs are also involved in NLRP-3 inflammasome activation. Other signaling pathways, including the TXNIP/NLRP-3/caspase-1 [83], Piezo 1/NLRP-3 [70], LINC00969/miR-335-3p/TXNIP (LINC00969 competitive endogenous RNA) [71], and cGAS/Sting/NLRP-3 axes (STING is an endoplasmic reticulum protein that stimulates the production of type I interferon; cGAS is a cytosolic DNA sensor that stimulates the synthesis of type I interferon) [84] are also associated with the regulation of inflammation activity through NLRP-3. In addition, there is increasing evience that NLRP-3 inflammasome targeting technology is a new HCMC strategy for IDD treatment. 

It should be recognized that so far knowledge about the mechanisms of NLRP-3 inflammasome activation in degenerating IVDs and about higher regulatory molecular pathways is limited. The study of the role of other inflammasomes in the development of IDD needs further study [85].

Melatonin inhibits the IL-1/NF-κB pathway and reduces mitochondrial reactive oxygen species (mtROS) in vivo and in vitro [86]. Additionally, it slows down IDD development by inhibiting the activation of inflammatory system via NLRP-3.

Administration of MCC-950 (as NLRP-3 inflammasome inhibitor) inhibited NLRP-3 inflammasome activation by *Acne propionibacteria* and reduced inflammatory response and apoptosis in degenerating IVDs in vivo [87]. In addition, fullerol nanoparticles can inhibit discogenic back pain by inhibiting NLRP-3 inflammasome activation [88,89,90].

However, targeted therapy still lacks selective inhibitors of NLRP-3 inflammasome, and the current use of targeted activity of NLRP-3 inflammasome in IDD treatment is limited to animal experiments [85].

## 6. Senolytics

The concept of senolytic therapy is that apoptosis can be selectively initiated in degenerating NP and AF cells by inhibiting survival mechanisms activated during aging. Zhu et al. [91] first demonstrated this concept by suppressing the very large B-cell lymphoma (BCL-XL) and EFNB1 survival pathways, which are highly activated by senescent cells, and other senolytic targets emerged [92,93,94]. During the degeneration of IVDs, a significant number of senescent cells are formed. The use of senolytics significantly inhibits the pathophysiological mechanism of the survival of old cells, which ultimately brings entire IVD tissue into an anti-aging state. This suggests that senolytics may be a new potential HTMC method for suppressing the active process of IVD cell degeneration, mitigating the symptomatic progression of IDD [44]. 

The study by Novais et al. [95] demonstrated that administration is a combination of senolytics (dasatinib and quercetin) on viability, phenotypic characteristics, and ECM structures in degenerative IVDs. Additionally, senolytics significantly prevented the progression of senescence of NP and AF cells in mouse IDD models. This combination of senolytics may be a new HTCM method for treatment of severe IDD [96]. Quercetin has similar anti-degenerative effects, which was demonstrated in an in vitro study [97]. These senolytic effects in IDD are potentially regulated through the NF-κB axis signaling pathway.

The senolytic ABT-263 is a formulation loaded into poly(lactic-co-glycolic acid) (PLGA-ABT) nanoparticles. A single intradiscal injection of PLGA-ABT can provide local delivery of the drug to avascular degenerating IVDs, prevent potential systemic toxicity caused by systemic senolytic administration, and morbidity caused by repeated drug injections into IVDs [98]. This HTMC method results in senescent cells elimination from degenerative IVDs, decreased pro-inflammatory cytokines overexpression, decreased MMPs expression, and inhibited IDD progression. Additionally, degenerative structure of IVD is restored [98].

Klotho senolytic reduces ECM degradation and neo-angiogenesis in IDD through inhibition of the Ras-1/PAK-1/MMP-2 signaling axis (by inhibition of Ras-associated substrate botulinum toxin C3 1 (Ras-1)/PAK-1 and MMP-2 protein expression by exogenous co-administration with Klotho with Ras-1 inhibitor) [99].

## 7. Exosomal Therapy

Exosomes are a type of extracellular vesicles that are 50–100 nm in diameter that originate from multivesicular endosomes. Exosomes are released by almost all cell types and contain various molecules (cytokines, lipids, proteins, and non-coding RNAs). Exosomes take part in intercellular communications, transferring their content between different cells of human tissues [100]. Transferred exosomal mRNA can be translated after entering another cell. It was proposed to name this type of RNA “exosomal shuttle RNA (esRNA)” [101].

Exosome secretion was shown to increase in response to inflammation, hypoxia, and an acidic microenvironment. Exosome secretion can be used for induction of immunosuppression and stimulation of neo-angiogenesis via miRNA delivery [102]. Delivery of miRNA by exosomes is contraindicated in cancer patients, but it may be useful in the treatment of IDD. It is assumed that exosomes restore damaged tissue and can maintain their therapeutic efficacy by transferring biologically active molecules and acting on target molecules that regulate gene expression and the phenotype of damaged recipient cells [101]. In addition, exosomes are able to survive under extreme conditions. They can confer resistance to oxidative stress in recipient cells [103].

Bone marrow-derived MSC-derived exosomes (BMSC-Exos) were found to reduce IL-1β-induced secretion of pro-inflammatory cytokines and activation of MAPK signaling by delivering miR-142-3p that targets mixed-origin kinase 3 (MLK-3) [104]. Exosomes derived from NP cells can not only induce differentiation of MSCs into NP-like cells in vitro, but also promote MSC migration and suppression of the Notch1 pathway [105]. NP cells from a rodent model of IVD hernia were found to produce miR-223-containing exosomes that suppress inflammation through modulation of the NF-κB pathway [106]. 

Exosomes derived from human placenta MSCs (hPLMSC) bearing AntagomiR-4450 (miR-4450 inhibitors) were tested for their therapeutic effect on mouse NP cells in vivo and in vitro. Inhibition of miR-4450 activates ZNF-121 (zinc finger protein 121), which facilitates inflammation, apoptosis, and damage to NP cells [107].

The exposure to doxorubicin significantly stimulated NLRP-3 expression, causing pyroptosis in H9c2 cells [108]. However, processing of exosomes derived from embryonic stem cells can inhibit pyroptosis in degenerating IVDs cells.

Lu et al. [109] believe that exosomes can be used as an alternative to MSC therapy in the treatment of IDD. They found that treatment with exosomes derived from MSCs had the same effect on NP cell proliferation as therapy with MSCs. Exosomes derived from MSCs prevent the progression of IDD, enhance antioxidant and anti-inflammatory effects in NP cells, or prevent apoptosis [110]. 

It is not known whether exosomes can regulate pyroptosis of NP cells during the progression of IDD. However, MSCs can inhibit NP cell pyroptosis by downregulating NLRP-3 inflammasome expression in a lipopolysaccharide-induced model. When MSCs were additionally treated with GW-4869 (neutral sphingomyelinase inhibitor) to inhibit exosome secretion, the antipyroptotic effect of the MSCs disappeared. This indicated that the effect of MSCs on pyroptosis may be caused by exosomes derived from them. Treatment with exosomes derived from MSCs significantly reduced the expression of the NLRP-3 inflammasome and decreased caspase activation, thereby suppressing the secretion of pro-inflammatory cytokines (IL-1β and IL-18) in NP cells upon stimulation with lipopolysaccharide [111].

It was shown that miR-410 regulates cell proliferation and apoptosis and acts as a prognostic biomarker in multifactorial inflammatory diseases [112]. Significantly elevated levels of miR-410 expression were reported to decrease the production of pro-inflammatory cytokines such as IL-10, TNF-α, IL-1β, and IL-6 [113]. Compared to healthy volunteers, miR-410 levels were reduced in patients with wet age-related macular degeneration [114]. MiR-410 levels in exosomes derived from MSCs were significantly higher than in exosomes derived from fibroblasts. These results indicate that miR-410 could be a potential mediator of NP cell pyroptosis. In addition, exosomal miR-410 derived from MSCs significantly inhibited the pyroptosis response by downregulating the NLRP-3/caspase-1 pathway in LPS-treated NP cells. Suppression of NLRP-3 by administration of exosomal miR-410 resulted in decreased levels of caspase-1 and GSDMD (gasdermin D), thereby attenuating pyroptosis of NP cells in degenerating IVDs. However, more evidence of an association between miR-410 and NLRP-3 is still required [111] in patients with IDD.

Exosomes in human umbilical cord MSCs (hucMSC) effectively improve NP cell viability and protect them from pyroptosis by targeting METTL-14 (methyltransferase-like protein 14) by methyltransferase, which catalyzes m6A modification. The METTL-14 protein is abundant in NP cells from IDD patients, which stabilize NLRP-3 mRNA in an IGFBP2-dependent manner (binding protein for IGF-I and -II). Such a pathogenic axis can be blocked by hucMSC exosomes, since these exosomes directly degrade METTL-14 via esRNA (for example, miR-26a-5p) [115].

Thus, cell scaffolds and exosome therapy are two new high-tech methods for correcting cytokine imbalances and degenerative processes in IVDs. The combination of these high technologies allows them to further enhance their therapeutic potential to stimulate proliferation and growth of NP cells, as well as their ability to regulate ECM metabolism in IDD development. This combination may help to more effectively inhibit this pathological process, in contrast to less successful classical therapeutic technologies for IDD [4]. Both ECM and exosomes have very low immunogenicity compared to cell therapy, which is an advantage for the clinical use of dECM@exo (EMC/exosomes) [4,116]. It is known that dECM@exo provides the best efficacy in IDD due to the synergistic effect of the extracellular matrix and exosomes and is more suitable for the IVD microenvironment [117,118]. Exosomes obtained from adipose-derived mesenchymal stem cell (ADSC) can slow down catabolism in degenerating IVDs by reducing the activity of MMPs. On the one hand, by slowing down the catabolism of the extracellular matrix, it is possible to correct the violation of its metabolism in IDD. On the other hand, dECM degradation is slowed down, allowing exosomes to remain in IVDs for up to 28 days. Storage of exosomes with dECM also allows exosomes to inactivate NLRP-3 inflammasome and inhibit pro-inflammatory cytokines overexpression in IVDs [116] in patients with IDD.

So, exosomes have the potential to be a more effective HTMC method for IDD in humans than MSCs-based [119]. Additionally, it is possible to genetically modify exosomes to express special ligands such as chemokine receptors that will better guide them to IVDs injury sites and deliver small molecule drugs directly to target sites, allowing exosomes to act as a targeted drug delivery mechanism [120]. While avascular nature of IVD poses a threat to the efficacy of systemically administered MSC-exosomes, attempts to extend their half-life may make this delivery method more popular [121]. 

## 8. Other Technologies

### 8.1. Factors Induced by Hypoxia

The hypoxia inducible protein (HIF) family consists of members of α- and β-subunits that come into action through formation of heterodimers under hypoxic conditions. HIF proteins have three distinct α-subunits (HIF-1α, HIF-2α, and HIF-3α) and one β-subunit, which is not affected by hypoxia. Heterodimers stimulate the expression of target genes by binding a consensus sequence called the hypoxia-sensitive element in the promoter regions [122].

HIF-2α activity is increased by hypoxia stimulation and TNF-α in NP cells, which depends on the duration of stimulation and dose. At the same time, TNF-α regulates hypoxia-induced erythropoietin expression, which is mediated primarily by HIF-2α rather than HIF-1α [123]. HIF-2α mediates the upregulation of MMP-13 and ADAMTS-4 by potentiating TNF-α/NF-κB signaling [124]. In turn, this plays an essential role in the regulation of the expression of genes that promote NP cell catabolism in degenerating IVDs. NF-κB protein is increased by TNF-α stimulation, while NF-κB inhibition reduces TNF-α-induced expression of HIF-2α protein and catabolic factors MMP-13 and ADAMTS-4 (A Disintegrin And Metalloproteinase 4) in degenerative IVD cells. Suppression of HIF-2α expression leads to slower degradation of the extracellular matrix, which may represent a new therapeutic strategy for IDD.

In addition, HIF-1α regulates the expression of type II collagen and aggrecan via the NOTCH-1 pathway in human NP cells. Receptors, ligands, and target genes of the NOTCH signaling pathway are expressed in NP and AF cells of degenerating IVDs, and the NOTCH signaling is critical for maintaining NP cell proliferation in hypoxic IVD niche [125]. However, the role of HIF-1α in regulation of cytokine balance in human IDD remains debatable.

### 8.2. Peptide NEMO-Binding Domain

It is known that the IDD process is mediated by NF-κB [4]. Mechanical compression of hNP/fibrin constructs leads to increased expression of MMP-3 and IL-8. Supplementation of the medium with 10 μM NEMO binding domain peptide (NBD) at the time of loading increased the viability of NP and AF cells and reduced expression levels of the *MMP3* gene. Damage to IVDs in rats resulted in increased expression of the *MMP3*, *IL1B*, and *IL6* genes. Injections of 250 μg NBD during IVD injury resulted in a decrease in the *IL6* gene overexpression and a decrease in the severity of the local inflammatory process.

The NBD peptide reduced exercise-induced levels of IL-1β and MMP-3 in hNPC/fibrin constructs, while increasing NP and AF cell viability as well as slowing down the rate of progression of IDD in rats, including down-regulation of IL-6. Therefore, NBD may be a potential HTMC method for correcting cytokine imbalance in IDD in humans [126].

### 8.3. Lysyl Oxidase

Lysyl oxidase (LOX) is an enzyme that in humans is encoded by the *LOX* gene. It catalyzes the conversion of lysine molecules into highly reactive aldehydes, which form crosslinks in ECM proteins in IVDs. LOX can protect chondrocytes from TNF-α-induced apoptosis and has an anti-apoptotic role in TNF-α-treated rat NP cells. This suggests that LOX may be a promising treatment for IDD. The *LOX* gene expression was significantly reduced in TNF-α-treated NP cells. Exogenous LOX can maintain TNF-α-induced activity of degenerating NP cells, decrease the rate of apoptosis, and stimulate EMC secretion in degenerating IVDs [127]. Additionally, LOX inhibits the Fas/FasL and p53 pathways [128], which play a role in the molecular mechanisms of IDD.

### 8.4. Cortistatin

Cortistatin (CST) is a cyclic neuropeptide that exhibits various properties in various physiological and disease processes [129]. Corresponding results in several autoimmune disease models, including inflammatory bowel disease and rheumatoid arthritis, showed that CST may be a natural endogenous anti-inflammatory factor [130]. In addition, CST is an attractive candidate for a new high-tech strategy for the treatment of degenerative and inflammatory conditions [131], including IDD. The recent study showed that CST improved metabolism, suppressed apoptosis, and attenuated inflammation in TNF-α-induced chondrocytes, and also combated articular cartilage degeneration in osteoarthritis [132].

NP cell apoptosis exacerbates the severity of IDD, and inhibition of NP cell apoptosis through the mitochondrial pathway may improve IDD. CST expression in NP cells decreases during aging and development of TNF-α-induced IDD. Exogenous CST treatment may reduce TNF-α mediated catabolism and apoptosis of degenerating IVDs. Furthermore, CST can inhibit mitochondrial dysfunction in NP cells and prevent IDD by targeting the activation of mitochondrial ROS-dependent NLRP3 inflammasomes [133].

### 8.5. Tenomodulin

Tenomodulin is a tendon/ligament-specific biomarker. It is an anti-angiogenic factor with abundant expression in IVDs. However, it is not yet clear whether tenomodulin contributes to the maintenance of homeostasis in degenerating IVDs by inhibiting vascular ingrowth into this normally avascular tissue. Tenomodulin acts as an inhibitor of angiogenesis in IVD homeostasis and protects against age-related degeneration of IVDs [134], making it promising for HTMC methods to correct the inflammatory response and apoptosis in IDD.

### 8.6. Bioactive Lipids 

Increased production of prostaglandin E2, a proinflammatory eicosanoid derived from arachidonic acid, is characteristic of degenerating NP and AF cells [135]. There is a hypothesis that a decrease in anti-inflammatory metabolites synthesis from polyunsaturated fatty acids may occur simultaneously. This hypothesis is supported by the observation that lipoxin A4 (LXA-4) has a significant effect on a rat model of non-compressible lumbar IVD hernia by inhibiting eicosapentaenoic acid, Janus kinase (JNK), and NF-κB/p65, reducing pro-inflammatory cytokines (IL-1β, TNF-α, etc.), as well as through the regulation of the expression of anti-inflammatory cytokines (TGF-β and IL-10) [136]. Interestingly, 14-epoxyeicosatetraenoic acid and 15-epoxyeicosatetraenoic acid (14,15-EET) derived from arachidonic acid protected rat NP cells from TNF-α-induced apoptosis in vitro by inhibiting the NF-κB pathway. Local administration of 14,15-EET prevented IDD progression [137]. Notably, a suite of soluble epoxide hydrolase enzymes can metabolize 14,15-EET and thus limit their beneficial effects, especially in the prevention of IDD. Therefore, inhibition of epoxide hydrolase enzymes to increase the half-life of EET may form a new approach to the prevention and treatment of IDD. Other studies [135,136,137] demonstrated that there is a delicate balance between pro-inflammatory prostaglandin E2 (and possibly other pro-inflammatory eicosanoids) and anti-inflammatory LXA-4 (and possibly resolvins, protectins, and maresins) and 14,15-EET (and other epoxyeicosatetraenes acids). If this balance could be shifted more towards LXA-4 and EET, then this could prevent cytokine imbalance in IDD. Interestingly, both prostaglandin E2 and LXA-4 (including resolvins, protectins, and maresins) downregulate overexpression of pro-inflammatory cytokines (IL-1β, IL-6, TNF-α) and MMPs and thus elicit their anti-inflammatory effects. Conversely, IL-1β, IL-6, and TNF-α increase COX-2 expression and enhance prostaglandin E2 and other pro-inflammatory eicosanoids production. 

The paradoxical effect of prostaglandin E2 to suppress IL-1β, IL-6, and TNF-α production, while having a pro-inflammatory effect, suggests that there is cross-talk between prostaglandin E2 and pro-inflammatory cytokines. This is intended to limit the inflammatory process in degenerating IVDs. Paradoxically, arachidonic acid forms both prostaglandin E2 and LXA-4 precursors. This means that regulation of the arachidonic acid pathway is critical in cytokine balance regulation in IVDs. It was reported that many inflammatory conditions in which increased prostaglandin E2 production are also characterized by arachidonic acid deficiency and decreased LXA-4 overproduction [138,139,140,141]. In these states of arachidonic acid deficiency, administration of arachidonic acid increases LXA-4 production without altering prostaglandin E2 synthesis [138,142].

Hyperglycemia induced by chemicals (alloxan and streptozotocin) in experimental animal models in type 1 and type 2 diabetes mellitus can be prevented by various n-3 and n-6 fatty acids, especially arachidonic acid and LXA-4 [143,144,145]. In addition, these experimental animals are noted to have low levels of arachidonic acid and LXA-4 in plasma and tissues [146,147] and elevated plasma levels of prostaglandin E2 [148]. Thus, it appears that both IDD and diabetes mellitus are inflammatory conditions, and both of them benefit from anti-inflammatory treatment strategies. This may explain why hyperglycemia (diabetes mellitus) exacerbates the severity of IDD, as shown in a study by Shan et al. [149].

### 8.7. Nanoparticles and Delivery Systems

Teixeira et al. [150] previously investigated anti-inflammatory nanoparticles. Chitosan (Ch)/Df/poly-γ-glutamic acid (γ-PGA) nanoparticles were able to inhibit and restore E2 prostaglandin production by activated macrophages in vitro. At the same time, it reduces overexpression of IL-6 and TNF-α [151]. The effect of these nanoparticles on the control of inflammation in IDD is being investigated. These nanoparticles were proven to be an effective drug delivery system that can be combined with other technologies.

Chitosan is a natural biodegradable polysaccharide. It is used for HTMC (drug delivery systems, gene therapy, and tissue engineering) [152]. Chitosan is nontoxic, biochemically active, and a biocompatible substance [153]. Chitosan and γ-PGA are ions with opposite charges that spontaneously self-assemble in a pH-controlled environment. Electrostatic interactions between chitosan and γ-PGA were previously studied [153]. Ch/γ-PGA polyelectrolytes are stable at pH 5.0 and were proposed as delivery systems for various proteins/molecules in various contexts: stromal factor-1 [154], IFN-γ [155], and Df (diclofenac) [154]. It was previously shown that Ch/γ-PGA nanoparticles with Df are an effective in vitro delivery system for anti-inflammatory drugs [154].

Intradiscal injection of Ch/Df/c-PGA nanoparticles reduced pro-inflammatory mediators (IL-6, IL-8, and prostaglandin E2) in a cell culture model of pro-inflammatory/degenerative IVDs. This anti-inflammatory delivery system also downregulated both MMP-1 and MMP-3 expression, but upregulated collagen II and aggrecan synthesis in degenerating IVDs. Ch/Df/c-PGA injection appears to be a promising intradiscal therapy for IDD. Moreover, the versatility of Ch/c-PGA nanoparticles allows them to be combined with other high-tech IDD therapies [150].

Also, co-administration nanoparticles (as an effective delivery system) with MSCs can be used for cytokine balance modulation [105] in patients with IDD.

## 9. Discussion

**Figure 6:**
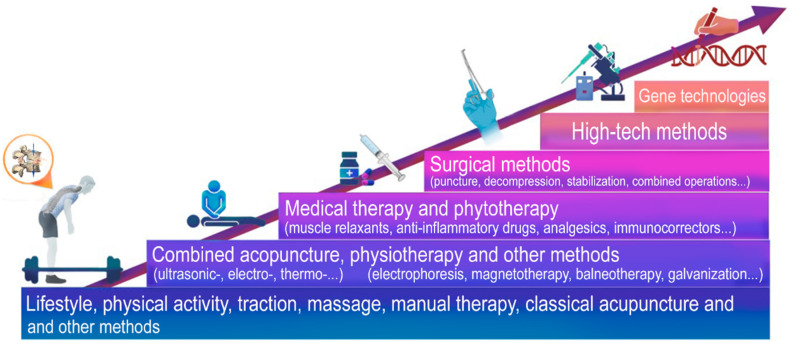
Evolution of approaches to the prevention and treatment of intervertebral disc degeneration in humans.

This narrative overview demonstrates the place of high technology in the evolution of treatments for IDD in humans (Figure 6).

The problem of cytokine imbalance correction in IDD is far from being resolved. This explains the need to develop new high-tech methods, since the available methods of treating IDD do not always give the expected therapeutic response. The total number and variety of methods for solving this problem are increasing. Recent researchers demonstrated the need to consider the IDD process at a deep pathophysiological level. At the same time, inflammation, as a typical pathological process, occupies a central position in the pathophysiological processes of IDD. Considering possible therapeutic options in terms of the pathophysiology of inflammation, the correction of cytokine imbalance in severe IDD by the HCMT methods presented in this narrative review is very promising (Table 1). These perspectives are based on the results of pre-clinical and clinical studies of the molecular mechanisms of these HCMT methods for IDD treatment.

**Figure 7:**
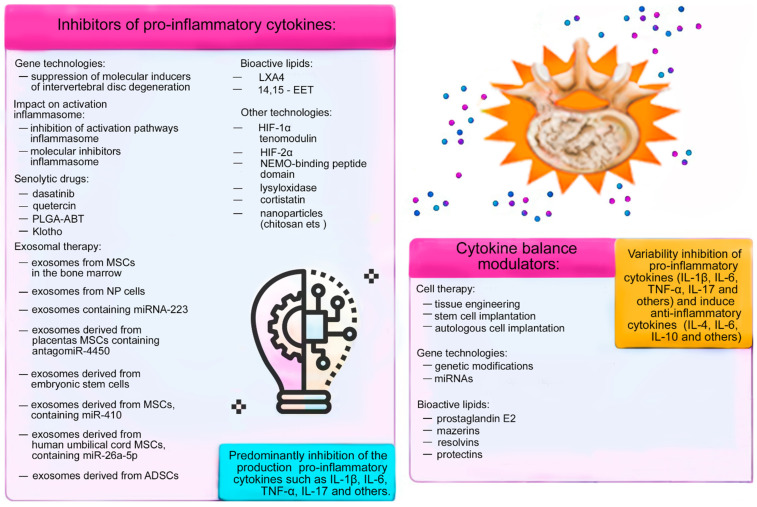
Molecular mechanisms of action of new high-tech methods for correcting cytokine imbalance in intervertebral disc degeneration (modified by the authors from Shnayder et al. [4]).

The HTMC methods presented in this review affect various molecular signaling pathways for the activation of the inflammatory response in IDD, which predominantly involves cytokines and other inflammatory mediators. Cell therapy, gene therapy, exosomal therapy, and other HTMC methods for correcting cytokine imbalance can slow down the progression of IDD and prevent the inflammatory response by reducing the level of pro-inflammatory cytokines or by increasing the level of anti-inflammatory cytokines. Such a therapeutic strategy would improve tissue regeneration and healing in damaged/degenerating IVDs by stimulating NP and AF cell proliferation, deposition of aggrecan, collagen, and other important molecules in the structures of IVDs (Figure 7).

These HTMC methods show potentially greater efficiency in the regulation of cytokine status in IDD, but require large economic and labor resources. However, stepwise IDD therapy (from symptomatic therapy for mild IDD to pathogenetic therapy for severe IDD) can help improve the expected therapeutic response, reduce the severity of chronic back pain, improve the quality of life of patients, and reduce the risk of disability.

## 10. Conclusions

This narrative review demonstrates progress in addressing chronic inflammation in general and cytokine imbalance, particularly in patients with IDD. New HTMC methods are directly or indirectly involved in the regulation of protective immune responses in the direction of reducing inflammation and cytokine imbalance, stimulating the regeneration of NP and AF cells of degenerating IVDs, blocking signaling pathways for the activation of pro-inflammatory cytokines, and their receptor overexpression. In addition, they can directly participate in the restoration of degenerative IVD structures. In the future, the use of these HTMC methods holds promise in cases of drug-resistant IDD and severe back pain. It is necessary to conduct further studies of the effectiveness and safety of these methods in real clinical practice.

## Figures and Tables

**Table 1:** High-tech medical technologies for cytokine imbalance correction in patients with intervertebral disc degeneration.

Method	Mechanism of Action	Evidence Class	References
I. Cell therapy			
Stem cell implantation	Suppression of pro-inflammatory cytokines synthesis (IL-6, IL-8, IL-5, IFN-γ, IL-1β, TNF-α, etc.). Supression of MMP-1 syntesis. Increased anti-inflammatory cytokines synthesis (IL-4, IL-10). Increased production of MMP-3 and TIMP-2, Tie-2-positive cells (clusters of differentiation). Prevention agaist cell death in degenerative IVDs. Reduction in proliferative response in degenerative IVDs. Increased deposition of agrecan and other IVD structures. Prevention of neoangiogenesis and neurogenesis in degenerating IVDs.	B	[10,11,12,13,14,23,25,26]
Implantation of autologous cells			
Tissue engineering			
II. Gene Technologies			
Gene modifications	Increased deposition of agrecan and other IVD structures. Overexpression of the *KLOTHO* gene (regulation of the TLR4-NF-KB signaling pathway). The *TRPV4* gene knockout (prevention of chronic vertebrogenic pain syndrome due to the influence of IL-6, IL-8). Introduction of the *FROB1* gene (increased proteoglycan production in degenerating IVDs). Introduction of the *LMP1* gene (increased expression of IL-4, IL-10, BMP-2, BMP-7, and aggrecan in degenerating IVDs). Introduction of the *COX9* gene (increased type II collagen synthesis in degenerating IVDs). Introduction of the *TIMP1* gene (increased proteoglycan content in degenerating IVDs). Introduction of the *GDF5* gene (increased synthesis of ECM structures in degenerating IVDs.	B	[3,35,36,37,38,40,41,42,43,44,45,46,47,48,58]
MicroRNA	Suppression of miR-34a (prevention of premature death of end plate chondrocytes and degradation of ECM structures through hyperproduction of IL-1β and repression of GDF-5). Suppression of miR-15b (prevention of overproduction of IL-1β by increasing SMAD-3 expression). Intoduction of miR-202-3p (suppression of IL-1β-induced MMP-1 production). Introduction of miR-10a-5p (inhibition of IL-6-induced ferroptosis of cartilage cells).	B	[56,59,60,61]
	Downregulation of miR-34a-5p (prevention of HOTAIR lncRNA binding and NP apoptosis by targeting NOTCH 1 receptors). Suppression of miR-153-3p in degenerating IVDs Prevention of LINC00641 binding (suppression of NP cell autophagy and NP cell death). Downregulation of miR-326 (LncRNA *SNHG1* overexpression promotes NP cell proliferation in degenerating IVDs). Introduction of miR-499a-5p (inhibition of NP cell apoptosis and ECM degradation in degenerating IVDs by targeting COX4). Suppression of the *XIST* gene expression in NP (prevention of cell apoptosis and EMC degradation by targeting miR-499a-5p). Regulation of the *TXNIP* gene transcription via LINC00969 (a competitive miR-335-3 and thereby regulating the activity of the NLRP3 inflammasome).	B	[65,66,67,68,69,71]
Molecular inductors	Suppression of LncRNA *TUG1* expression (protection against TNF-α-induced apoptosis and senescence by blocking the Wnt/β-catenin pathway). Suppression of LncRNA *ZFAS1* expression (reduction in the risk and severity of IDD). Suppression of LncRNA *HOTAIR* expression contributes (prevention of aging and apoptosis of NP cells, as well as degradation of EMC structures via Wnt/β-catenin activation). Suppression of the *XIST* gene expression contributes (prevention of IL-1β-induced degeneration of NP cells). Introducrion PIEZO1-siRNA (suppression of *Piezo1* expression, inhibition of Ca/NF-κB pathway activation in the NLRP-3 inflammasome, decrease in inflammatory response in degenerating IVDs).	C	[62,63,64,65,70]
III. Technologies for Influencing Inflammatory Cascade in Degenerative Intervertebral Discs Mediated by Abnormal Inflammasome Activation			
Inflammasome inhibitor NLRP-3 MCC-950 + Propionibacterium acne	Inhibition of NLRP-3 inflammasome activation. Reduction in inflammatory response in degenerating IVDs. Reduction in apoptosis in degenerating IVDs.	B	[87]
Fullerol nanoparticles	Inhibition of NLRP3 inflammasome activation (decrease in vertebrogenic back pain).	B	[88]
IV. Senolytics			
Combination of dazatinib (Src/tyrosine kinase inhibitor) + Quetercin (natural flavonoid)	Binding to BCL-2. Modulation of transcription factors, cell cycle proteins, pro- and anti-apoptotic proteins, growth factors and protein kinases. Inhibition of pathophysiological survival mechanisms of old cells in degenerating IVDs.	C	[44,95,96,97]
PLGA-ABT	Binding to BCL-2. Modulation of transcription factors, cell cycle proteins, pro- and anti-apoptotic proteins, growth factors and protein kinases. Inhibition of pathophysiological mechanisms of survival of old cells in degenerating IVDs. Inhibition of hyperproduction of pro-inflammatory cytokines (IL-1β, IL-6, and TNF-α). Restoration of degenerated IVDs structures.	C	[44,98]
Senolytic Klotho	Binding to BCL-2. Modulation of transcription factors, cell cycle proteins, pro- and anti-apoptotic proteins, growth factors and protein kinases. Inhibition of pathophysiological mechanisms of survival of old cells in degenerating IVDs. Inhibition of the Rac1/PAK1/MMP-2 signaling axis. Reduction in IVDs structures degradation. Inhibition of neoangiogenesis in degenerated IVDs.	C	[44,99]
V. Exosomal Therapy			
Exosomes derived from bone marrow MSCs (BMSC-Exos)	Inhibition of IL-1β-induced of pro-inflammatory cytokines overexpression (IL-1β and IL-18). Activation of MAPK signaling by miR142-3p delivery, which targets MLK-3. Inhibition of pyroptosis associated with NLRP-3 expression in degenerated IVDs.	B	[104,110]
Exosomes originating from NP cells	Induction of differentiation of MSCs into NP-like cells. Stimulation of MSCs migration. Inhibition of the NOTCH-1 pathway.	B	[105]
Exosomes containing miR-223 isolated from cells of the NP model of rodent herniation	Modulation of the NF-κB pathway. Inhibition of inflammatory in degenerating IVDs.	B	[106]
Exosomes derived from hPLMSC bearing antago-miR-4450 (miRNA-4450 inhibitors)	Inhibition of miR-4450. Activation of ZNF-121. Reduction in inflammation in degenerating IVDs. Reduction in apoptosis in degenerating IVDs. Reduction in NP cells degradation.	B	[107]
Exosomes derived from embryonic stem cells	Inhibition of pyroptosis associated with NLRP3 overexpression in degenerating IVDs.	B	[108]
Exosomal miR-410 derived from MSCs	Inhibition of pyroptosis by suppression of the NLRP3/caspase-1 pathway (decrease in caspase-1 and GSDMD levels). Inhibition of pro-inflammatory cytokines overexpession (IL-10, TNF-α, IL-1β, and IL-6).	C	[111,113]
Exosomes of hucMSC and miR-6a-5p	Protection of IVDs cells from pyroptosis by targeting METTL-14 required. Stabilization of NLRP-3 mRNA in an IGFBP2-dependent manner by methyltransferase catalyzing m6A modification.	B	[115]
Exosomes derived from ADSC	Inhibition of MMPs activity. Inactivation of NLRP3 inflammasome. Inhibition of pro-inflammatory cytokines release (IL-1β, TNF-α, and IL-6).	C	[116]
VI. Other Technologies			
Factors induced by hypoxia	Inhibition of HIF-2α expression. Retardation of degenerative processes in IVDs. Upregulation of MMP-13 and ADAMTS-4 by potentiation of TNF-α/NF-κB signaling.	C	[123,124]
	Inhibition of HIF-1α. Regulation of expression of collagen type II and aggrecan via the NOTCH-1 pathway in degenerating IVDs.	C	[125]
NEMO-binding domain	Increased cell viability of degenerating IVDs by inhibition of the *MMP3* gene expression. Decrease in MMP-3 and pro-inflammatory cytokines (IL-1β, IL-6) levels in degenerating IVDs.	C	[126]
Lysyl oxidase	Protection of chondrocytes from apoptosis induced by TNF-α. Inhibition of the Fas/FasL and p53 89 pathway involved in degenerating IVDs.	C	[127,128]
Cortistatin	Improvement of metabolism. Reduction in apoptosis in degenerating IVD. Reduction in inflammatory in TNF-α-induced chondrocytes. Inhibition of mitochondrial dysfunction by targeting the activation of mitochondrial ROS-dependent NLRP-3 inflammasomes in degenerating IVDs.	C	[131,132,133]
Tenomodulin	Inhibition of neoangiogenesis in degenerating IVDs. Protection against age-related degeneration of IVDs.	C	[134]
VII. Bioactive Lipids			
LXA-4	Inhibition of eicosapentaenoic acid, JNK and NF-kB/p65. Inhibition of IL-1β, TNF-α, and other pro-inflammatory cytokines.	C	[136]
14,15-EET	Protection of NP cells from TNF-α-induced death. Inhibition of the NF-κB pathway.	B	[135,136,137]
Prostaglandin E2	Inhibition of pro-inflammatory cytokines overexpression (IL-1β, IL-6, TNF-α). Decrease in MMPs levels in degenerating IVDs.	C	[135,136,137]
Nanoparticles and delivery systems	Chitosan nanoparticles (Ch/Df/γ-PGA) inhibit and restore prostaglandin E2 production by activated macrophage. Reduction in production of IL-6, IL-8 and partially TNF-α. Suppression of MMP-1 and MMP-3 overexpression. Upregulation of collagen II and aggrecan synthesis in degenerating IVDs. Ensuring an effective delivery system of anti-inflammatory drugs in IDD.	C	[150,151,154]

Note: 14,15-EET—14 epoxyeicosa-tetraenoic acid and 15 epoxyeicosa-tetraenoic acid; ADSC—adipose-derived mesenchymal stem cell; Ch—chitozane; COX-1—cyclooxeginase 1; IDD—intervertebral disk degeneration; IFN-γ—interferone gamma; IL-1β—interleukine 1 beta; IL-5—interleukine 5; IL-6—interleukine 6; IL-8—interluekine 8; GSDMD—gasdermin D; hucMSC—human umbilical cord mesenchymal stem cells; hPLMSC—human placenta mesenchymal stem cells; JNK—Janus nucleus kinase; LXA-4—lipoxin A4; MSCs—mesenchymal stem cells; MMP-1—metalloproteinase 1; MMP-3—metalloproteinase 3; MLK-3—mixed origin kinase 3; NP—nucleus pulposus; PLGA-ABT—ABT263—drug loaded into poly(lactic-co-glycolic acid) nanoparticles; TNF-α—tumor necrosis factor alpha; γ-PGA—gamma-polyglutaric acid; and ZNF-121—zinc finger protein 121.

## Data Availability

Not applicable.
